# The effects of thylakoid-rich spinach extract and aqueous extract of caraway (*Carum carvi* L.) in letrozole-induced polycystic ovarian syndrome rats

**DOI:** 10.1186/s12906-020-03044-w

**Published:** 2020-08-12

**Authors:** Saeed Sherafatmanesh, Maryam Ekramzadeh, Nader Tanideh, Mohammad-Taghi Golmakani, Farhad Koohpeyma

**Affiliations:** 1 Student Research Committee, School of Nutrition and Food Sciences, Shiraz University of Medical Sciences, Shiraz, Iran; 2 Nutrition Research Center, Department of Clinical Nutrition, School of Nutrition and Food Sciences, Shiraz University of Medical Sciences, Shiraz, Iran; 3 Stem Cell and Transgenic Research Center, Shiraz University of Medical Sciences, Shiraz, Iran; 4 Department of Food Science and Technology, School of Agriculture, Shiraz University, Shiraz, Iran; 5 Shiraz Endocrinology and Metabolism Research Center, Shiraz University of Medical Sciences, Shiraz, Iran

**Keywords:** Ovarian cysts, Insulin resistance, Chlorophyll, Ovarian function tests, Tumor necrosis factor-alpha, Phytotherapy, Blood glucose, Luteinizing hormone, Body weight

## Abstract

**Background:**

Polycystic ovary syndrome (PCOS) is a common endocrine disorder. The aim of the present study was to evaluate the effects of the oral administration of thylakoid-rich spinach extract and the caraway aqueous extract in letrozole-induced polycystic ovary syndrome rats**.**

**Methods:**

Sixty female Sprague-Dawley rats were randomly divided into five groups of 12 animals each. Letrozole (1 mg/kg) was administered orally for a period of 28 days to induce PCOS. Sham and PCOS control rats received 1 mL/day of distilled water, and the three groups of PCOS rats were given 150 mg/kg of metformin, 3 g/kg of caraway, and thylakoid at a dose of 6 mg chlorophyll/gr food intake/day by oral gavage for 8 weeks. Finally, blood samples were collected and the right ovary of rats was removed, weighed, and fixed in 4% buffered formalin to determine the biochemical and stereological parameters.

**Results:**

Compared to the PCOS control group, consuming metformin, thylakoid, and caraway extracts significantly improved the fasting blood sugar (FBS), tumor necrosis factor-alpha (TNF-α), malondialdehyde (MDA), luteinizing hormone (LH), insulin resistance, and body weight, increased the volume of the corpus luteum, and reduced the number of atretic follicles after 8 weeks (푃< 0.05). Although caraway treatment caused a significant increase in the HDL-C (High-density lipoprotein cholesterol) level (*P* < 0.001), no significant change was observed in terms of HDL-C in the thylakoid and metformin groups compared to the PCOS control group.

**Conclusion:**

Our data showed that the consumption of thylakoid and caraway extracts for 8 weeks may have beneficial effects on the biochemical and stereological factors in PCOS-induced rats.

## Background

Polycystic ovary syndrome (PCOS) is the most common endocrine and metabolic disorder among women, affecting 6.5–8.0% (based on the NIH 1990 criteria) of women during their reproductive years worldwide [1]. It is characterized based on the Rotterdam Criteria including polycystic ovarian, ovulatory abnormality (oligo−/anovulation), and hyperandrogenism [2]. PCOS is the leading cause of infertility and increases adverse pregnancy outcomes in reproductive-aged women [3]. PCOS patients exhibit a cluster of risk factors for the development of type 2 diabetes and cardiovascular disease, including insulin resistance, hyperglycemia, lipid profile disorders, abdominal obesity, and other related diseases [4]. The mutual relationship between central obesity and insulin resistance has been considered as the most characteristic symptom of PCOS [5]. Also, free radicals generated during long-term hyperglycemia impose a burden of oxidative stress on the body [6]. Currently, PCOS treatment is based on pharmacological agents such as clomiphene citrate, tamoxifen, metformin, and glucocorticoids; however, the efficacy of these therapies is limited due to their major side effects, including psychological perturbation, muscle pain, nausea, and diarrhea [7, 8]. Hence, finding natural alternatives with fewer adverse effects is necessary in order to overcome the negative health impacts which are associated with PCOS.

Thylakoids are chlorophyll-containing membranes in chloroplasts isolated from green leaves such as spinach. They consist of protein-bound pigments, including chlorophyll, beta-carotene, lutein, and zeaxanthin, and antioxidants, such as carotenoids and vitamin E [9]. According to both animal experimental models and human studies, the oral intake of thylakoids can promote satiety, decrease blood lipids, reduce fasting blood glucose level, and cause a notable body weight loss [10]. The antiobesity and antihyperlipidemic activities of thylakoid have been highlighted in the literature due to the point that it can prolong dietary fat digestion, thereby increasing the secretion of satiety signals, such as cholecystokinin (CCK), and down-regulating the hunger signals, such as ghrelin [10, 11].

Caraway (*Carum carvi* L.), from the Apiaceae family, is one of the well-known herbs, naturally found in Iran, Northern and Central Europe, and Turkey [12]. It contains phytochemicals, including carvone and limonene, which have high potential antioxidant properties [12]. The tolerability and hepatoprotection of caraway have been approved by previous studies [13]. Earlier reports have shown the ability of caraway intake to improve the lipid profile and lower the glucose level in the body [12]. The aqueous extracts from the roots of caraway plants have been shown to have high antioxidant activity, which can have a crucial role in modulating the formation of atherosclerotic plaque [14]. Also, it is noteworthy to mention that caraway could suppress the sense of hunger via the same underlying mechanisms triggered by thylakoids, such as higher secretion of CCK hormone resulting in body weight loss [15]. Therefore, in the present study, it was hypothesized that the oral intake of thylakoid-rich spinach extract and aqueous extract of caraway could be associated with the attenuation of PCOS complications.

## Methods

### Preparation of thylakoid extract

All of the required fresh baby spinach leaves (*Spinacia oleracea*) were harvested on March 2017 from a farm located in the Shiraz region, Fars province, Iran (29°36′49.5“ N latitude, 52°28’00.3” E longitude). Spinach leaves were frozen directly after harvest and kept so until the extraction procedure. Plant taxonomy expert (Dr. khosravi) from the Department of Biology, College of Sciences, Shiraz University, Shiraz, Iran, were asked to identify the genus and they verified it with the herbarium number of 2617. At first, 5 kg of spinach leaves and 5 l of water were homogenized in a blender (cutter Alexanderwerk 65, Poland) for 10 min, followed by a filtration step to remove large cell debris and non-disrupted cells. The obtained smooth green slurry was then filtered through two-layer filter papers and a 20-μm mesh (Whatman International Ltd. Maidstone, England, Cat No. 1441–125). The filtrate was poured into centrifugal tubes (250 ml) and centrifuged for 20 min at 6084×g (SW 14R, Froilabo, Meyzieu, France). The supernatant was discarded and the thylakoids in the pellet were collected and re-suspended with 5 ml distilled water and stored in falcon conical-bottom disposable plastic tubes at − 20 °C until the beginning of the experiment. We conducted laboratory procedures regarding the determination of quantity of chlorophyll for our thylakoid-rich spinach extract via spectrophotometry method according to Ostbring et al. [16]. Determining the content of chlorophyll content is one method to measure the amount of thylakoid membranes [17]. Thirty ml thylakoid extract was added to 2 ml ice-cold acetone (80 vol, %). The samples were then incubated dark and centrifuged at 25 °C for 4 min at 12100×g. The chlorophyll a, b and total content was determined at the absorbance of 646.6 nm and 663.6 nm by a spectrophotometer (Varian Inc., Santa Clara, CA, USA).

### Preparation of caraway aqueous extract

Dry seeds of caraway were acquired from a local herbal market (Attari Shojaee market, March 2017, Shiraz, Iran) and the identity of the genus (herbarium number of 2603) was certified by plant taxonomy expert (Dr. khosravi) from the Department of Biology, College of Sciences, Shiraz University, Shiraz, Iran. Caraway seeds were cleaned and powdered with a home blender. Then, 400 g of powdered seeds was mixed with 1600 ml of distilled water and blended together. Afterward, the mixture was stirred using a magnetic stirrer (Labinco model L81, DG Breda, Netherlands) for 72 h, 300 rpm. The supernatant was separated and the residual content was centrifuged at 5000 rpm, 10 min and stored frozen at − 20 °C in falcon conical-bottom disposable plastic tubes until the time of the experiment.

### Experimental animals

The protocols of the study were approved by the Institutional Animal Ethics Committee (IAEC) of Shiraz University of Medical Sciences (Shiraz, Iran), following the NIH guidelines for the care and use of animals (NIH publication NO. 85–23, revised in 1996). Initially, 60 non-pregnant female Sprague-Dawley rats (aged 10–12 weeks and weighing 180-220 g) were acquired from the Laboratory Animals Research Center (Shiraz University of Medical Sciences, Iran). Before starting the experiment, the animals were allowed to acclimatize to the laboratory environment for 2 weeks. During the study, all the rats were fed with chow diet (Pars Dam Co., Tehran, Iran) and water ad libitum. They were caged in stainless steel cages and maintained in a temperature-controlled (22–25∘C) environment with 50 ± 5% humidity and a 12 h light/dark cycle. The animal procedures in this experiment were conducted in accordance with the ethics stated in the Guide for the Care and Use of Laboratory Animals [18].

### Induction of PCOS

Before the induction of PCOS, vaginal smears were collected from all animals for 2 weeks to evaluate the regularity of their reproductive cycle. The induction of the disease was done during the estrus phase of the estrous cycle. All the experimental animals, except the sham group, were orally administered with an emulsion of 1 mg/kg letrozole (Aburaihan Pharma Co., Tehran-IRAN) which had been dissolved in normal saline for 28 consecutive days [19, 20]. Subsequently, a microscopic evaluation of the collected vaginal smears was conducted to confirm the induction of PCOS.

### Study design

In the present study, animals were divided randomly into five equal groups of 12 rats each:

- **Sham group:** Non-PCOS induced rats that received 1 mL/day distilled water;
- **PCOS control group:** PCOS-induced rats that received 1 ml/day distilled water;
- **Metformin group:** PCOS-induced rats that received metformin (Shafa Pharmaceutical Co., Tehran, IRAN) dissolved in normal saline at a level of 150 mg/kg body weight/day. Given that the dose of metformin used in the previous articles were 150 mg/kg and 300 mg/kg, in this study we decided to consider the dose of 150 mg/kg [21, 22].
- **Caraway group:** PCOS-induced rats that received caraway aqueous extract at a level of 3 g/kg body weight/day;
- **Thylakoid group:** PCOS-induced rats that received thylakoid extract at a level of 6 mg chlorophyll/gr food intake/day (with an average of 20 g food/day) [11]. It is worth mentioning that the amount of chlorophyll content was proportional to the concentration of thylakoids based on the existing evidence. Thus, the treatment dose of thylakoid can reflect the chlorophyll content of the extract [17].

All treatments were by oral gavage for 8 weeks.

### Collection of blood samples

At the end of the intervention, the rats were fasting for 12 h. Then, they were euthanized using a chamber pre-filled with carbon dioxide (CO2) gas with a concentration of 70% which, based on the previous studies, is a common and safe method for euthanizing laboratory rats [23]. Approximately, 5 mL of blood was collected by cardiac puncture and centrifuged at 3500 rpm for 10 min to separate the serum. The serum was used for the evaluation of study parameters. All the serum samples were stored in clean sterile micro-centrifuge tubes at − 80 ∘C until further analysis.

### Biochemical measurements

The serum concentrations of FBS (fasting blood sugar), triglyceride (TG), total cholesterol (TC), LDL cholesterol (LDL-C), and HDL cholesterol (HDL-C) were assayed using an enzymatic colorimetric method, a biochemical autoanalyzer (BT-1500, Italy), and a commercial diagnostic kit (Pars Azmoon Co., Tehran, Iran). An ELISA kit (Bioassay Technology Laboratory, Cat No. E0707Ra) was used to determine the serum insulin concentrations. HOMA-IR was calculated for each rat using the following equation: HOMA-IR = Fasting insulin (μU/mL) × Fasting glucose (mg/dL)/405 [24]. The level of MDA (malondialdehyde) was measured spectrophotometrically with the thiobarbituric acid reactive substances (TBARS) method [25]. Serum TNF-α (tumor necrosis factor-alpha) level was measured using the ELISA kit (IBL International, BE45471) according to the manufacturer’s instructions. Serum LH (luteinizing hormone) and FSH (follicle-stimulating hormone) were determined via ELISA kits (Bioassay Technology Laboratory, Cat No. E1037Ra and E0182Ra, respectively).

### Tissue preparation and stereological measurements

Based on previous studies, the rate of ovulation, volume of corpus luteum, and ovarian weight are higher on the right ovary than left side in mouse [26]. Thus, the right ovary of the rats was removed, weighed, and fixed in 4% buffered formalin to ensure the same condition for collecting our stereological data. After tissue processing, the samples were blocked in cylindrical paraffin blocks. Serial 5- and 20 μm-thick sections were obtained using a microtome and stained with hematoxylin and eosin (H&E) (Merck Company, Germany) method.

**Fig. 1:**
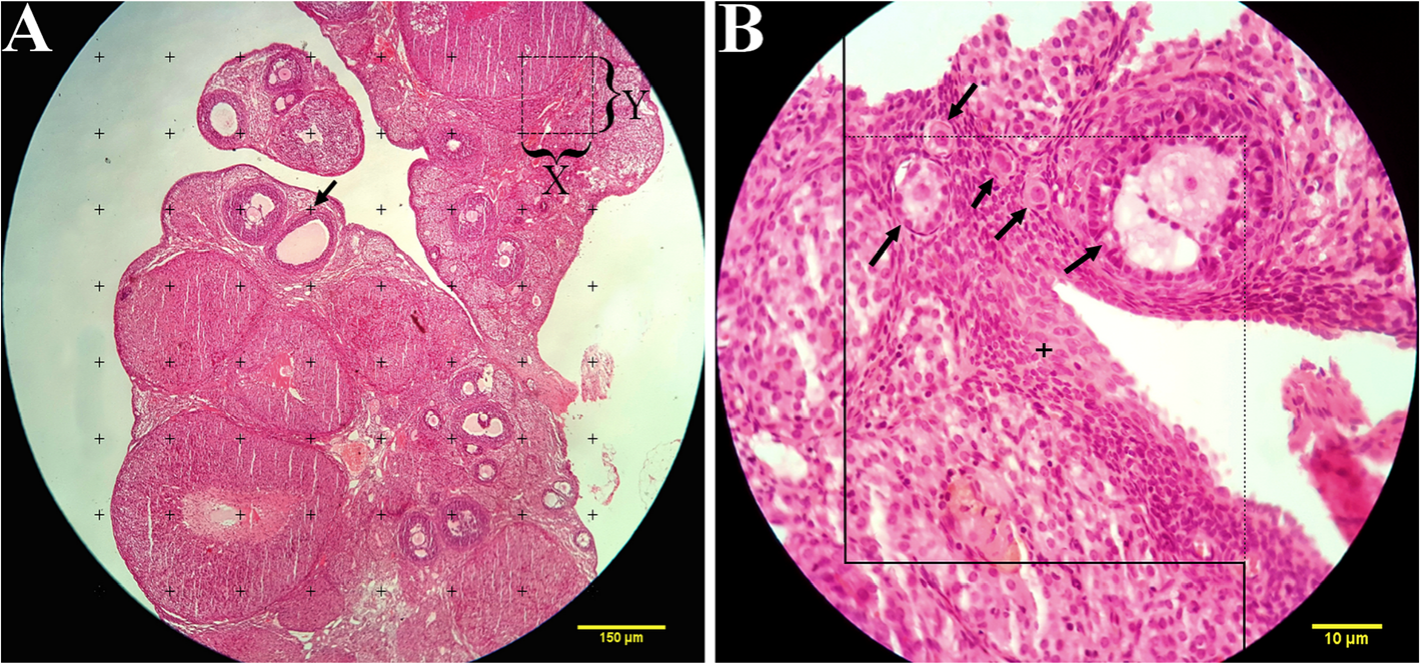
**a** Point-counting method to estimate the volume density of the cortex, medulla, cystic formation, and corpus luteum. The arrow indicates the right upper quadrant of each cross that was considered to be a point. **b** The optical disector method. An unbiased counting frame was laid on the images. The follicle oocytes were counted if they placed completely or partly inside the counting frame, touched the upper and right lines (acceptance line), or did not touch the left and lower borders (rejection lines) (arrows). Upper and lower guard zones were defined, and each of them was set to be 5 μm using a Heidenhain microcator (MT12, Germany)

The orientator method was used to obtain isotropic uniform random (IUR) sections [27]. The Cavalieri method was applied to estimate the total volume of the ovary. From each ovary, 8–12 sections were sampled randomly. Counting probe was randomly placed on the images; then, the total number of points hitting the sections was counted (Fig. 1a). The total volume of the ovary was estimated using the following formula:

$$ {V}_{total\ ovary=}\sum \limits_{i=1}^np\times a(p)\times t $$

In this formula, ‘${\sum}_{i=1}^np$’ is the total number of points superimposed on the image, ‘a (p)’ the area associated with each point, and ‘t’ the distance between the sample sections.

The volume density of the targeted structure (here cortex, medulla, corpus luteum, and ovarian cysts) was estimated on 5 μm-thick sections through the point-counting method and using Delesse formula [19] (Fig. 1a):

$$ Vv(structure)=\sum \limits_{i=1}^np\ (structure)/\sum \limits_{i=1}^n(reference) $$

In which ‘${\sum}_{i=1}^np(structure)$’ is the number of the test points falling on the targeted structure (here cortex, medulla, corpus luteum, and ovarian cysts) and ‘${\sum}_{i=1}^np$ (reference)’ is the total points hitting the ovary sections. The following formula was used to estimate the absolute targeted structure volumes [28]:

$$ V(structure)=V(ovary)\times Vv(structure) $$

The number of follicles was determined on 20 μm-thick sections using an optical disector method (Fig. 1b) and the following formula:

$$ Nv=\frac{\sum_{i=1}^nQ}{\sum_{i=1}^nP\times h\times \left(\frac{a}{f}\right)}\times \frac{t}{BA} $$

In this formula, ‘${\sum}_{i=1}^nQ$’ is the number of the follicles counted in all of the dissectors, ‘h’ the height of the optical disector, ‘a/f’ the area of the counting frame, ‘${\sum}_{i=1}^nP$’ the total number of the counted frames, ‘BA’ (block advance) the setting of the microtome to cut the paraffin block, and ‘t’ the mean of the final section thickness [28]. To estimate the total number of the follicles, the following formula was applied:

$$ {\mathrm{N}}_{\left(\mathrm{Follicles}\right)}={\mathrm{N}}_{\mathrm{V}\left(\mathrm{follicles}/\mathrm{ovary}\right)}\times {\mathrm{V}}_{\left(\mathrm{ovary}\right)} $$

### Statistical analysis

The statistical analysis was performed using SPSS (version 22.0). The normal distribution of the data was assessed using the Kolmogorov–Smirnov test. One-way ANOVA, followed by LSD multiple range post-hoc test, was used to compare the study groups in terms of the mean differences in the serum and stereological parameters. Repeated measures ANOVA was used to compare the mean body weight between groups at different measurement times. For all of the study markers, at first a comparison between the sham group and PCOS control group were done to be sure that markers in the PCOS control group is different significantly with the sham group. Then, we compared the study parameters between the treated groups and the PCOS control group. All the differences were considered significant at *P* ≤ 0.05.

## Results

### Body weight changes

**Fig. 2:**
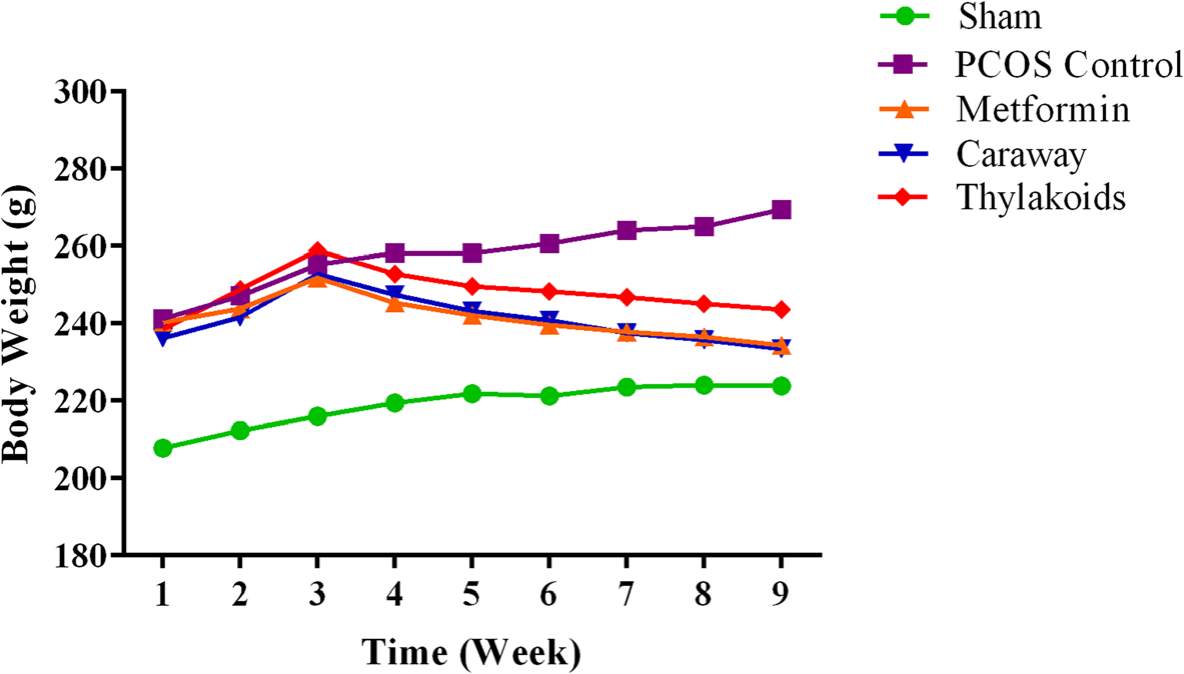
The trend of body weight changes in each group fed with different treatments

The rats were monitored weekly for body weight from the beginning to the end of the experiment. At the beginning of the intervention, the PCOS-induced rats showed a significant increase in body weight compared to the sham group (*P* < 0.05). The week-group interaction showed that during the intervention period, the body weight changes between the study groups were statistically significant. In fact, the body weight notably increased in the PCOS control group while the treated rats showed a significantly lower body weight compared to the PCOS control group (*P* < 0.001) (Table 1) (Fig. 2).

**Table 1:** Body weights change of each group fed with different treatments

Group	Pre-intervention	Week 1	Week 2	Week 3	Week 4	Week 5	Week 6	Week 7	Week 8	***P***-value		
										Week	Group	Week ∗ group
**Sham**	207.83 ± 12.7	212.36 ± 13.43	216.09 ± 13.75	219.45 ± 11.79	221.9 ± 11.32	221.27 ± 12.55	223.54 ± 12.16	224.09 ± 11.57	223.9 ± 11.61	0.003*	0.002*	< 0.001*
**PCOS control**	241.25 ± 16.2	247.18 ± 13.85	255.27 ± 12	258.27 ± 10.77	258.18 ± 9.4	260.63 ± 9.28	264 ± 8.24	265 ± 8.78	269.45 ± 8.76			
**Metformin**	240.33 ± 21.64	243.8 ± 22.38	251.9 ± 20.61	245.3 ± 18.58	242.2 ± 17.1	239.7 ± 16.6	237.8 ± 16.07	236.5 ± 16.02	234.4 ± 17.01			
**Caraway**	236.25 ± 20.91	241.63 ± 21.36	252.9 ± 21.22	247.45 ± 21.6	243.27 ± 21.16	240.81 ± 20.39	237.54 ± 21.8	235.72 ± 21.28	233.45 ± 22.75			
**Thylakoids**	238.5 ± 27.9	248.81 ± 32.48	258.9 ± 34.45	252.81 ± 34.17	249.63 ± 33.69	248.36 ± 33.69	246.9 ± 34.5	245.18 ± 34.24	243.63 ± 32.81			

*Sham* Non PCOS induced rats that received distilled water, *PCOS control* PCOS induced rats that received distilled water, *Metformin* PCOS induced rats that received metformin, Caraway PCOS induced rats that received Caraway aqueous extract, *Thylakoids* PCOS induced rats that received thylakoid extract

Data expressed as Mean ± SD, all differences were considered significant at *P* ≤ 0.05

### Glycemic parameters

According to the serum insulin concentrations, there was no statistically significant difference between any of the study groups (*p* = 0.36). Both HOMA-IR and FBS levels exhibited a significant increase (*P* < 0.001) in the PCOS control group in comparison to the sham group. All of the treated rats showed a significant decrease in the FBS level (*P* < 0.001) when compared to the rats in the PCOS control group. Also, there was a significant reduction in the level of HOMA-IR parameter in the thylakoid group (*P* = 0.003), caraway group (*P* = 0.006), and metformin group (*P* = 0.001) (Table 2).

**Table 2:** Serum parameters after 8 weeks of intervention

Parameter	Sham	PCOS control	Metformin	Caraway	Thylakoids	*P*- value
**TG (mg/dl)**	40.81 ± 5.07^a^	63.72 ± 3.25^b^	48.6 ± 4.9^c^	46.45 ± 4.76^c^	49 ± 3.16^c^	< 0.001*
**TC (mg/dl)**	41.45 ± 1.96^a^	64.81 ± 3.15^b^	50.3 ± 4.13^c^	48.72 ± 4.1^c^	49.9 ± 2.94^c^	< 0.001*
**LDL_C (mg/dl)**	14.27 ± 1.1^a^	23.18 ± 2.22^b^	16.5 ± 1.5^dc^	15.27 ± 2^ad^	16.81 ± 0.98^c^	< 0.001*
**HDL_C (mg/dl)**	21.18 ± 2.27^a^	13.18 ± 0.87^b^	13.9 ± 1.19^b^	16.09 ± 1.04^c^	13.9 ± 0.83^b^	< 0.001*
**FBS (mg/dl)**	95.9 ± 4.52^a^	151.27 ± 11.76^b^	115.3 ± 3.65^c^	118.36 ± 4.43^c^	119.27 ± 3.43^c^	< 0.001*
**Insulin (mIU/L)**	11.1 ± 3.79^a^	14.88 ± 3.76^a^	12.15 ± 4.29^a^	13.39 ± 6.14^a^	12.96 ± 3.74^a^	0.36
**HOMA − IR**	2.62 ± 0.90^a^	5.51 ± 1.33^b^	3.48 ± 1.28^ac^	3.92 ± 1.8^c^	3.8 ± 1.04^c^	< 0.001*
**TNFα (pg/ml)**	103.60 ± 35.13^a^	148.62 ± 21.95^b^	115.11 ± 24.10^a^	107.55 ± 41.19^a^	105.75 ± 32.85^a^	0.04*
**MDA (pg/ml)**	2.29 ± 0.1^a^	3.19 ± 0.2^b^	2.6 ± 0.28^c^	2.64 ± 0.18^c^	2.52 ± 0.28^c^	< 0.001*
**LH (mIU/mL)**	9.96 ± 3.01^a^	18.96 ± 4.29^b^	13.53 ± 2.97^a^	14.14 ± 4.31^a^	14.04 ± 7.21^a^	0.003*
**FSH (IU/mL)**	17.86 ± 3.45^a^	22.85 ± 9.61^a^	23.19 ± 7.32^a^	22.36 ± 5.77^a^	22.44 ± 7.17^a^	0.37

*Sham* Non PCOS induced rats that received distilled water, *PCOS control* PCOS induced rats that received distilled water, *Metformin* PCOS induced rats that received metformin, Caraway PCOS induced rats that received caraway aqueous extract, *Thylakoids* PCOS induced rats that received thylakoid extract, *TG* Triglyceride, *TC* Total Cholesterol, *LDL_C* Low density lipoprotein, *HDL_C* High density lipoprotein, *FBS* Fasting blood sugar, *HOMA−IR* Homeostatic model assessment-insulin resistance, *TNFα* Tumor necrosis factor alpha, *MDA* Malondialdehyde, *LH* Luteinizing hormone, *FSH* Follicle-stimulating hormone

Data expressed as Mean ± SD, In each row, figures bearing different superscripts are significantly different at *p* < 0.05 (one way ANOVA)

### Serum lipid profile

The effects of treatments on the serum lipids are shown in (Table 2). TGs, TC, and LDL-C levels significantly increased (*P* < 0.001) while the HDL-C level significantly decreased (*P <* 0.001) in the PCOS control group compared to that of the sham group. There was a significant decrease in the concentrations of TGs, TC, and LDL-C in all the treated rats compared to those in the PCOS control group (*P <* 0.001). Although caraway treatment caused a significant increase in the HDL-C level (*P <* 0.001), there was no statistically significant difference between the thylakoid and metformin groups and the PCOS control group in terms of the HDL-C level.

### Anti-inflammatory and antioxidant parameters

By the end of the study, a significant increase (*P* = 0.005) was observed between the PCOS control group and the sham group in terms of the serum TNF-α concentration. A statistically significant difference was observed between the Metformin-, caraway-, and thylakoids-treated rats compared to the PCOS control group in terms of the serum TNF-α level (*P* = 0.039, *P* = 0.012, and *P* = 0.011, respectively). The serum MDA level was significantly increased (*P* < 0.001) in the PCOS control group when compared to that of the sham group. Compared to the PCOS control group, Metformin, caraway, and thylakoids treatments notably decreased the MDA level (*P* = 0.001, *P <* 0.001, and *P <* 0.001)), respectively)) (Table 2).

### Serum LH and FSH hormones

Serum LH concentration significantly increased in the PCOS control group compared to that of the sham group (*P <* 0.001). Animals treated with metformin, caraway, and thylakoids showed a significant decrease in the LH levels (*P* < 0.023, *P* < 0.035, and *P* = 0.032, respectively) in comparison with those in the PCOS control group. No statistically significant difference was observed in the serum FSH concentrations in any of the study groups (*P* = 0.37) (Table 2).

### Stereological study

#### Ovarian weight and volume parameters

**Fig. 3:**
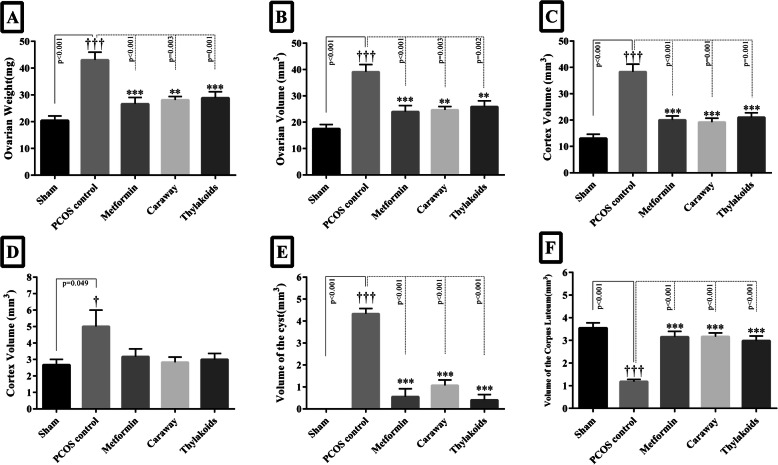
The column graph of weight (**a**), ovary volumes (**b**), cortex (**c**), medulla (**d**), cysts (**e**), and corpus luteum (**f**). Significant differences are exhibited in each column. All differences were considered significant at *P* < 0.05. † *p* < 0.05, PCOS vs. sham group; * *p* < 0.05, PCOS vs. metformin, caraway, and thylakoid groups. * *p <* 0.05, † *p* < 0.05. ** *p* < 0.01, †† *p* < 0.01. *** *p* < 0.001, ††† *p* < 0.001

**Fig. 4:**
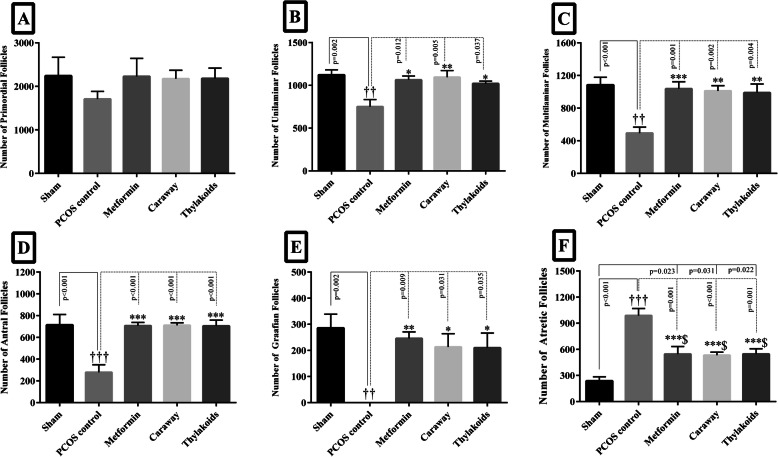
The Column graph of the number of primordial (**a**), unilaminar (**b**), multilaminar (**c**), antral (**d**), Graafian (**e**), and atretic (**f**) follicle oocytes. Significant differences are exhibited in each column. All differences were considered significant at *P* < 0.05. † *p* < 0.05, PCOS vs. sham group; * *p <* 0.05, PCOS vs. metformin, caraway, and thylakoid groups; $ *p* < 0.05 sham vs. metformin, caraway, and thylakoid groups. * *p* < 0.05, ** *p* < 0.01, *** *p* < 0.001, † *p* < 0.05, †† *p* < 0.01, ††† *p* < 0.001, $ *p* < 0.05

**Fig. 5:**
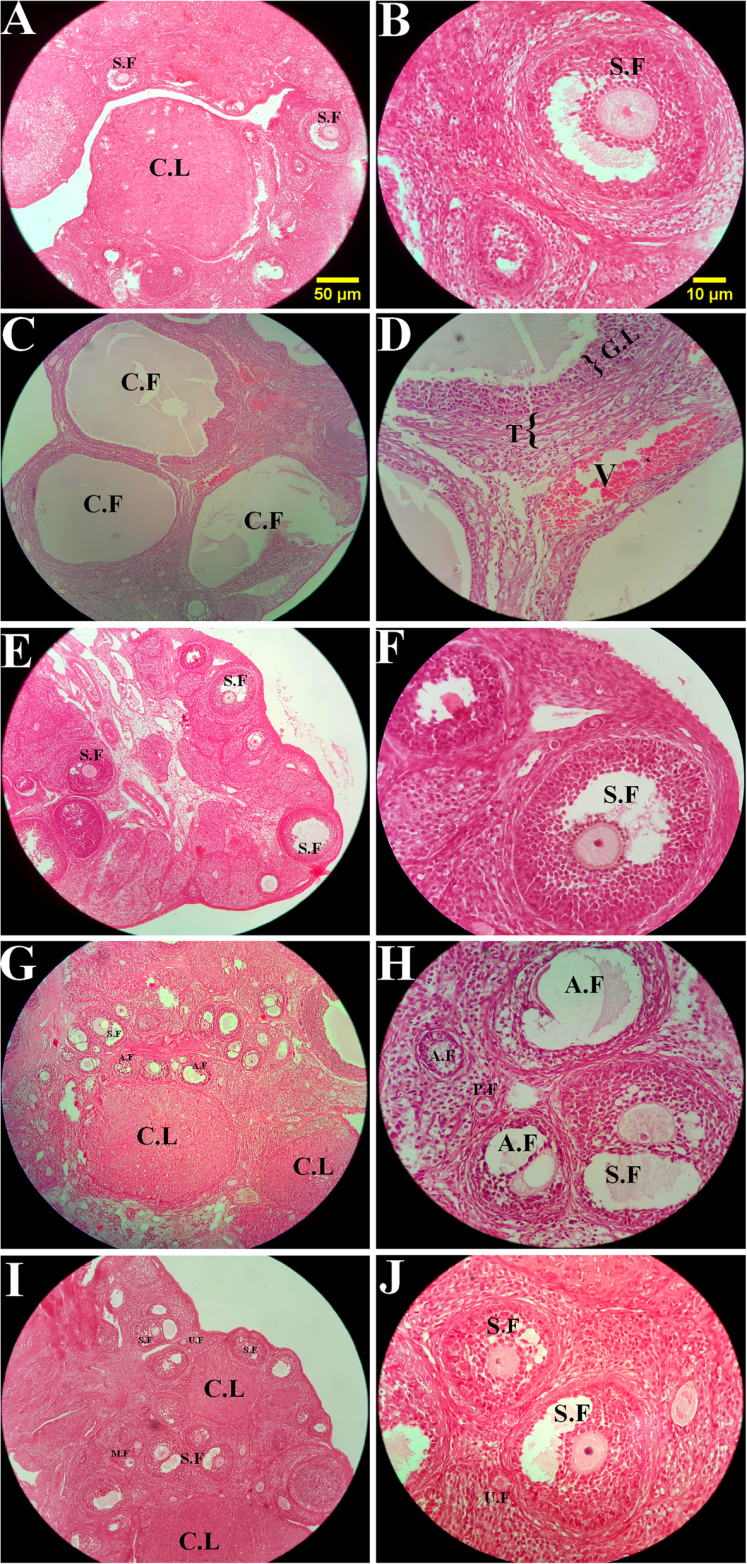
The comparison of the microscopic images of the ovaries in sham group (**a**, **b**), PCOS control (**c**, **d**), metformin (**e**, **f**), caraway (**g**, **h**), and thylakoids (**i**, **j**). S.F: Secondary (antral) follicle, C.L: Corpus luteum, C.F: Cyst follicle, A.F: Atretic follicle, P.F: Primordial follicle, U.F: Unilaminar follicle, M.F: Multilaminar follicle, G.L: Granulosa layer, T: Theca layer. H&E staining with magnification at (**a**, **c**, **e**, **g**) × 100, and (**b**, **d**, **f**, **h**) × 400. **a**, **b**: The different developmental stages of folliculogenesis were normal in the sham group. **c**, **d**: The PCOS control group showed a significant increase in the number of atretic follicle oocytes and volume of the ovarian cyst along with a decreased in the volume of the corpus luteum. **e-j:** All treated groups showed a noticeable reduce in the number of ovarian cysts along with higher volume of the corpus luteum and healthy follicles. However, the number of atretic follicle oocytes in these groups was still significantly higher in comparison to the sham group

The effects of treatments on stereological parameters are shown in (Figs. 3, 4, 5 a-h). The weight and volume of the ovary and the volume of the cortex, medulla, and cyst significantly increased (*P* < 0.05) while the volume of the corpus luteum significantly decreased (*P* < 0.001) in the PCOS control group compared to the volumes recorded in the sham group (Fig. 3a-f). Compared to the PCOS control group, all the treatment groups showed a significant decrease in the weight and volume of the ovary and the volume of the cortex and cyst (*P* < 0.05), accompanied by a significant increase in the volume of the corpus luteum (*P* < 0.001) (Fig. 3a, b, c, e, f). However, there was no statistically significant difference between the treatment groups and the PCOS control group regarding the ovarian volume of the medulla (Fig. 3d).

#### Total number of the ovarian follicles

According to the results of the stereological evaluation, there was no significant difference between the study groups in terms of the number of primordial follicles (Fig. 4a). The number of the unilaminar, multilaminar, antral, Graafian follicles significantly decreased (*P* < 0.05), while the number of atretic follicles significantly increased (*P* < 0.001) in the PCOS control group compared to those of the sham group (Fig. 4b-f). A significant increase was observed in the number of unilaminar, multilaminar, antral, Graafian follicles (*P <* 0.05), along with a significant decrease in the number of atretic follicles, in all treated animals in comparison to the numbers recorded for those in the PCOS control group (*P* < 0.001) (Fig. 4b-f). However, the reduction in the atretic follicle numbers due to metformin, caraway, and thylakoids treatments was not as pronounced as that in the sham group (*P* = 0.02, *P* = 0.03, and *P =* 0.02, respectively) (Fig. 4f).

## Discussion

To the best of our knowledge, this study was the first effort to investigate the effects of the oral intake of caraway and thylakoid in letrozole-induced PCOS rat models.

In the present study, the letrozole-aromatase inhibitor was used to induce polycystic ovary syndrome in the study animals. According to the earlier studies, the histology of ovaries taken from letrozole-treated animals have remarkable similarities to human PCOS features than to other PCOS models [29]. In this pathway, both the formation of large ovarian cysts and atretic follicles and the biochemical disorders occurred, which is in accordance with earlier studies [30]. PCOS is known as a metabolic disorder and has been closely associated with the features observed in the metabolic syndrome, such as insulin resistance, obesity, oxidative stress, dyslipidemia, and inflammation [4]. In the current study, a considerable increase was observed in the FBS level in the PCOS control group, which is consistent with the findings of Maharjan et al.’s study on hyperglycemia in the letrozole-induced PCOS rat models [31]. The oral administration of caraway and thylakoid significantly reduced the FBS and HOMA-IR parameters in the PCOS-induced animals, a finding which is in agreement with the results of earlier studies [11, 32]. It appeared that the underlying mechanisms via which caraway exerted its hypoglycemic effect had deterrent effects on both the hepatic glucose production and renal glucose reabsorption or intestinal glucose absorption. Higher glucose utilization by peripheral tissues altered enzymatic pathways by, for instance, decreasing the activities of gluconeogenic enzymes and increasing the activities of glycolytic enzymes [32, 33]. In addition, it is likely that the hypoglycemic mechanism of thylakoids is activated by an increase in the activities of glucose-metabolizing enzymes, such as glucose-6-phosphate dehydrogenase [34]. Furthermore, as highlighted earlier, thylakoids are one of the most concentrated sources of beta-carotene [9]. Ford et al. reported that all serum carotenoids, especially beta-carotene, were inversely related to hyperglycemia and insulin resistance [35]. Therefore, it can be argued that caraway and thylakoids may act as antihyperglycemic agents in PCOS patients due to the mechanisms described above. In addition, there was no significant difference in the basal plasma insulin concentration after treatment with both thylakoid and caraway extracts, indicating that the underlying pathways could be independent of insulin secretion.

In the present study, a significant weight gain, together with an impaired lipid profile, was observed in the PCOS control group in comparison with the sham group, a finding which is in agreement with the results of Kandarakis et al.’s study [36]. Our findings showed that the intake of caraway and thylakoids results in a significant body weight loss and improvement in TGs, TC and LDL-C parameters when compared to the PCOS control group. There are convincing reports about the mechanisms by which caraway can exert its lipid-lowering and antiobesity effects. These features of caraway may be relevant to its polyphenolic constituents, such as carvone and limonene [37]. It has been reported that carvone and limonene, which also exist in the *Anethum graveolens* extract, can cause a significant reduction in the serum TC and LDL-C levels [38]. Additionally, in their study, Stefania et al. showed the antihyperlipidemic activity of quercetin, which is a subset of caraway flavonoids [39]. These bioactive components may also reduce the serum cholesterol and modify the metabolism of the serum lipoprotein by reducing the activity of 3-hydroxy-3-methyl-glutaryl-coenzyme A reductase, which plays a key role in the biosynthesis of cholesterol, and enhancing the uptake of LDL through increasing LDL receptors [40]. Furthermore, caraway fibers may bind to the bile acid within the intestine and delay the dietary fat absorption by increasing bile acid excretion [32]. On the other hand, it has been documented that the presence of phenolic compounds and galactolipids in thylakoids can lead to a prolonged dietary fat digestion by inhibiting the pancreatic lipase-colipase complex and preventing the subsequent lipolysis in the intestine, thereby triggering the ‘ileal brake’ and the release of the appetite-suppressing hormones [41]. The ‘ileal brake’ is potentially an excellent long-term target for appetite regulation and sustainable weight management; it is defined as an important inhibitory feedback in the digestive tract to control the passing of a meal so as to ensure the improvement in digestion and absorption of nutrients [42]. The stimulation of ‘ileal brake’ causes gut cells to release appetite-suppressing hormones, such as CCK and glucagon-like peptide-1 (GLP-1), into the bloodstream [42, 43]. It is noteworthy to mention that ‘ileal brake’ has an important role in the improvement of glycemic control [42]. Fullness sensation and increased level of anorexigenic hormones, including cholecystokinin, glucagon-like peptide-1, and leptin, have been observed after the intake of meals enriched with thylakoids [9]. Moreover, thylakoids are resistant to degradation by gastric and pancreatic enzymes, allowing their advantageous effects to last longer [44]. Moreover, in a study done by Köhnke et al., dual-energy X-ray analysis revealed a body fat reduction in the female mice after treatment with thylakoids [17].

In the present study, a significant increase was observed in the concentration of both serum TNF-α and MDA in the PCOS control rats. An increase in the levels of inflammation and oxidative stress, together with the production of reactive oxygen species (ROS), have been reported to occur in PCOS [45]. In addition, the role of TNF-α and ROS in the pathogenesis of ovarian cysts, the increase in the number of ovarian atretic follicles, and the decrease in the volume of corpus luteum has been reported in previous studies [46, 47]. Animals fed with caraway and thylakoids exhibited lower levels of TNFα and MDA. According to earlier studies, the presence of polyphenolic compounds in caraway exhibited a high antioxidant activity, which subsequently resulted in a reduced level of free radicals in the body [48]. Moreover, carvone, as the most well-known anti-inflammatory agent in caraway, has been reported to inhibit the activity of 5-lipoxygenase and cyclooxygenase enzymes, which, in turn, can reduce the burden of inflammation due to the lower biosynthesis of leukotrienes and prostaglandins in the body [48]. As highlighted earlier, the antioxidant content of thylakoids, such as chlorophylls, flavonoids, and carotenoids, may be helpful in alleviating the oxidative stress by scavenging the ROS [49]. Moreover, chlorophyll derivatives may stimulate peroxisome proliferator-activated receptors (PPARs), which are involved in regulating various metabolic pathways, particularly the anti-inflammatory reactions [50]. In addition, thylakoids are among the most concentrated sources of vitamin E, which possesses anti-inflammatory and antioxidant properties [51]. Eventually, it can be stated that caraway and thylakoids may be helpful in treating ovarian disorders due to their anti-inflammatory and antioxidative stress activities.

Elevated levels of LH and the modest or unchanged concentration of FSH have been reported to occur in PCOS [52, 53]. Venturoli et al. reported a positive relationship between LH level and higher volumes of ovarian atretic follicles, a finding which is in agreement with our stereological results [54]. Animals treated with caraway, and thylakoids showed a significant decrease in the serum LH levels in comparison to the PCOS control group. In one study, Nasri et al. showed a notable decrease in the serum LH concentrations in rats treated with the Vitex agnus castus L. extract [55]. Also, Bates et al. reported a positive correlation between the body weight loss and reduced levels of androgens, which subsequently led to a reduction in the LH concentration and the restoration of cyclic ovulation in obese and infertile women [56]. Hence, in this study, we assumed that the improvement in the serum LH concentration occurred due to the active phenolic constituents in both caraway and thylakoid extracts and their antiobesity properties, which could have an affirmative effect on the ovarian volume and the number of ovarian follicle.

The limitations of this study should be taken into account when interpreting the results. As a result of limited funding, we could not investigate and evaluate other precious biochemical and molecular indices, such as serum androgens, blood leptin, adiponectin. Also, some important molecular parameters, including the expression of steroidogenic-related genes/proteins and proliferative markers, were not evaluated in this study and that could affect the reliability of our results. Therefore, future studies need to evaluate other specialized markers to clarify the other beneficial effects of caraway and thylakoid intake.

## Conclusion

In conclusion, the results of this study suggest that the use of caraway and thylakoid extracts can be considered as a plausible phytotherapeutic approach for the better management of biochemical and ovarian complications in the PCOS rat models.

## Data Availability

The datasets used and/or analysed during the current study are available from the corresponding author upon request.

## Abbreviations

PCOS: Polycystic ovary syndrome
FBS: Fasting blood sugar
TNF-α: Tumor necrosis factor-alpha
MDA: Malondialdehyde
LH: Luteinizing hormone
FSH: Follicle-stimulating hormone
IAEC: Institutional Animal Ethics Committee
TG: Triglyceride
TC: Total cholesterol
LDL-C: Low-density lipoprotein cholesterol
HDL-C: High-density lipoprotein cholesterol
IUR: Isotropic uniform random
ANOVA: One-way analysis of variance
CCK: Cholecystokinin
GLP-1: Glucagon-like peptide-1
ROS: Reactive oxygen species
PPARs: Peroxisome proliferator-activated receptors
